# Bis(2-meth­oxy­benzyl­ammonium) di­aqua­bis­(di­hydrogen diphosphato-κ^2^ *O*,*O*′)manganate(II) dihydrate

**DOI:** 10.1107/S1600536813026366

**Published:** 2013-10-02

**Authors:** Adel Elboulali, Samah Akriche, Mohamed Rzaigui

**Affiliations:** a Laboratoire de Chimie des Matériaux, Faculté des Sciences de Bizerte, 7021 Zarzouna Bizerte, Tunisia

## Abstract

The asymmetric unit of the title compound, (C_8_H_12_NO)_2_[Mn(H_2_P_2_O_7_)_2_(H_2_O)_2_]·2H_2_O, consists of half an Mn^II^ complex anion, a 2-meth­oxy­benyl­ammonium cation and a solvent water mol­ecule. The Mn^II^ complex anion lies across an inversion center, and has a slightly distorted octa­hedral coordination environment for the Mn^II^ ion, formed by two bidentate dihydrogendiphosphate ligands and two water mol­ecules. In the crystal, the components are linked by O—H⋯O and N—H⋯O hydrogen bonds, forming layers parallel to (100). An intra­molecular N—H⋯O hydrogen bond is also observed.

## Related literature

For related structures, see: Alaoui Tahiri *et al.* (2003 ▶); Selmi *et al.* (2006 ▶, 2009 ▶); Ahmed *et al.* (2006 ▶); Gharbi *et al.* (1994 ▶); Gharbi & Jouini (2004 ▶); Elboulali *et al.* (2013 ▶). For valence-sum calculations, see: Brown & Altermatt (1985 ▶).
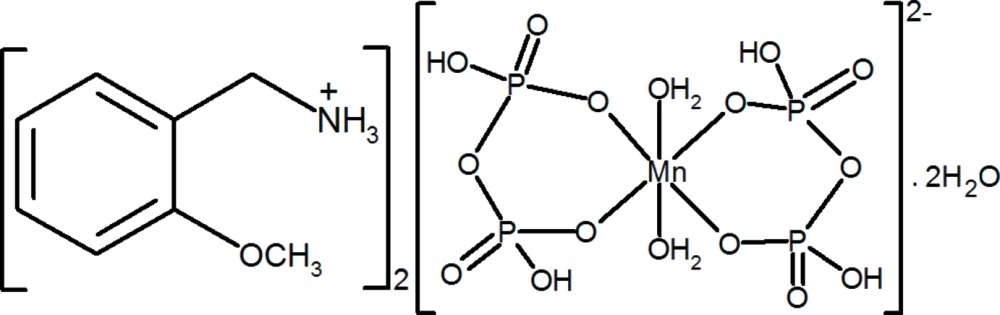


## Experimental

### 

#### Crystal data

(C_8_H_12_NO)_2_[Mn(H_2_P_2_O_7_)_2_(H_2_O)_2_]·2H_2_O
*M* *_r_* = 755.29
Monoclinic, 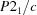

*a* = 13.971 (2) Å
*b* = 12.150 (3) Å
*c* = 9.169 (2) Å
β = 93.80 (4)°
*V* = 1553.0 (6) Å^3^
*Z* = 2
Ag *K*α radiation
λ = 0.56087 Å
μ = 0.37 mm^−1^
*T* = 293 K
0.3 × 0.2 × 0.1 mm

#### Data collection

Enraf–Nonius CAD-4 diffractometer
Absorption correction: multi-scan (Blessing, 1995 ▶) *T*_min_ = 0.920, *T*_max_ = 0.933
9935 measured reflections
7513 independent reflections
4417 reflections with *I* > 2σ(*I*)
*R*_int_ = 0.028
2 standard reflections every 120 min intensity decay: −1%

#### Refinement

*R*[*F* ^2^ > 2σ(*F* ^2^)] = 0.047
*wR*(*F* ^2^) = 0.112
*S* = 0.98
7513 reflections
212 parameters
6 restraints
H atoms treated by a mixture of independent and constrained refinement
Δρ_max_ = 0.98 e Å^−3^
Δρ_min_ = −0.45 e Å^−3^

### 

Data collection: *CAD-4 EXPRESS* (Enraf–Nonius, 1994 ▶); cell refinement: *CAD-4 EXPRESS*; data reduction: *XCAD4* (Harms & Wocadlo, 1996 ▶); program(s) used to solve structure: *SHELXS97* (Sheldrick, 2008 ▶); program(s) used to refine structure: *SHELXL97* (Sheldrick, 2008 ▶); molecular graphics: *ORTEP-3 for Windows* (Farrugia, 2012 ▶) and *DIAMOND* (Brandenburg & Putz, 2005 ▶); software used to prepare material for publication: *WinGX* (Farrugia, 2012 ▶).

## Supplementary Material

Crystal structure: contains datablock(s) I. DOI: 10.1107/S1600536813026366/lh5654sup1.cif

Structure factors: contains datablock(s) I. DOI: 10.1107/S1600536813026366/lh5654Isup2.hkl

Additional supplementary materials:  crystallographic information; 3D view; checkCIF report

## Figures and Tables

**Table 1:** Hydrogen-bond geometry (Å, °)

*D*—H⋯*A*	*D*—H	H⋯*A*	*D*⋯*A*	*D*—H⋯*A*
O1—H1*O*1⋯O3^i^	0.82	1.77	2.5689 (17)	164
O5—H5*O*5⋯O7^ii^	0.82	1.76	2.5711 (18)	169
O1*W*—H1*W*1⋯O3^iii^	0.86 (1)	1.98 (1)	2.8304 (19)	174 (3)
O1*W*—H2*W*1⋯O7^ii^	0.85 (1)	2.04 (1)	2.879 (2)	167 (2)
O2*W*—H1*W*2⋯O7^iii^	0.86 (1)	2.03 (1)	2.886 (2)	175 (3)
O2*W*—H2*W*2⋯O3^ii^	0.85 (1)	2.05 (1)	2.8842 (19)	164 (2)
N1—H1*N*1⋯O2*W*	0.89	1.97	2.826 (2)	160
N1—H2*N*1⋯O6^iv^	0.89	2.06	2.826 (2)	144
N1—H3*N*1⋯O8	0.89	2.45	2.991 (2)	120
N1—H3*N*1⋯O2	0.89	2.27	2.957 (2)	134

Symmetry codes: (i) 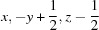; (ii) 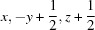; (iii) 
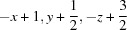
; (iv) 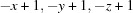.

**Fig. 1.**
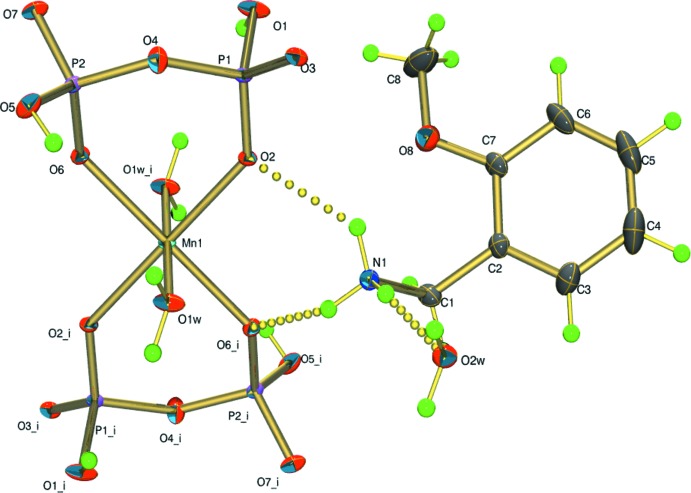
The molecular structure of (I). Displacement ellipsoids are drawn at the 30% probability level. H atoms are represented as small spheres of arbitrary radii. Hydrogen bonds are represented as dotted lines [Symmetry code: (i) 1 - x, 1 - y, 1 - z]. Only the symmetry unique cation and solvent water molecule are shown.

**Fig. 2.**
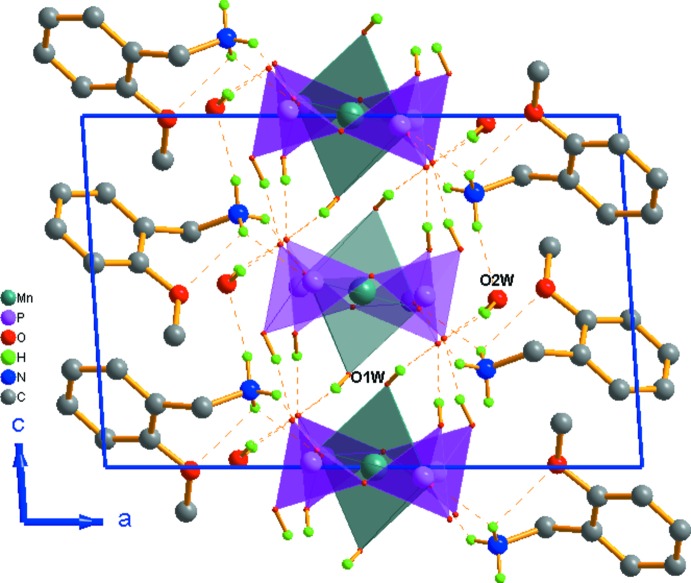
Crystal packing of (I) viewed along the b-axis. The H-atoms not involved in H-bonding are omitted.

## supplementary crystallographic information

### 1. Comment

As a part of our study of crystal packing in diphosphate materials, a new hybrid compound of mixed organic-metal cations have been synthesized: (C_8_H_12_NO)_2_[Mn(H_2_P_2_O_7_)_2_(H_2_O)_2_]·2H_2_O (I).

The asymmetric unit of (I) is made up of a half of mononuclear [Mn(H_2_P_2_O_7_)_2_(H_2_O)_2_]^2-^ moiety, one of organic cation and one water of crystallization. As the Mn^II^ ion lies on inversion centre, the complete formula unit is generated by this element of symmetry (Fig. 1).

In the crystal packing, each Mn^II^ ion is coordinated by two bidentate diphosphate ligands and two coordinated O1W water molecules to form a slightly distorted MnO_6_ octahedron. The valence bond calculation (Brown & Altermatt, 1985) gives an effective bond valence of 1.9417 consistent with the cationic charge of +2.

The Mn—O bond distances around the Mn^II^ ion are in the range 2.1389 (12)–2.1987 (14) Å which is close to those reported for Mn metal in K_2_Mn(H_2_P_2_O_7_)_2_·2(H_2_O) framework (mean Mn—O = 2.173 Å) (Alaoui Tahiri *et al.*, 2003), and slightly longer compared to those around Co in related structures (Selmi *et al.*, 2006,2009; Ahmed *et al.*, 2006). The P_2_O_7_ ligand has a quasi-eclipsed conformation with O—P—P—O torsion angles averaging 19.5 ° and it bridges the Mn^II^ ion through O2—P1 and O6—P2 linkages thus producing a bent P_2_O_7_ group, with a P1—O4—P2 angle of 134.68 (9)° as observed in other M(II)–organic diphosphate frameworks (Selmi *et al.*, 2006, 2009; Ahmed *et al.*, 2006; Gharbi *et al.*, 2004;1994). On the other hand the main bond lenghts of organic cations, are comparable to those observed in the *4*-methoxybenzylammonium cations in the (C_8_H_12_NO)_2_(H_2_P_2_O_7_) structure reported earlier (Elboulali *et al.*, 2013).

The MnO_6_ octahedra are arranged into anionic layers spreading along *a*-axis at *x* = 1/2 (Fig.2) *via* O—H···O hydrogen bonding interactions involving the hydroxyl groups of [H_2_P_2_O_7_]^2-^ and OW1 water molecules. The remaining uncoordinated O2W water molecules and *2*-methoxybenzylammonium cations further link these so as to contribute to their cohesion with O···O and N···O separations ranging from 2.826 (2)to 2.991 (2) Å (Table 1) and build a two-dimensionnal network parallel to (100).

### 2. Experimental

Crystals of the title compound were synthesized by the reaction of diphosphoric acid H_4_P_2_O_7_ (2 mmol), MnCl_2_·4H_2_O (0.2 g; 1 mmol) and *2*-methoxybenzylamine (0.138 g; 1 mmol) carried out in an acidic medium. The diphosphoric acid, H_4_P_2_O_7_, was obtained from Na_4_P_2_O_7_ by using an ion-exchange resin (Amberlite IR 120).

### 3. Refinement

All H atoms attached to C, O and N atoms were fixed geometrically and treated as riding, with C—H = 0.93 Å with *U*_iso_(H) = 1.2U_eq_(C) for the aromatic ring and C—H = 0.97 and 0.96 Å and N—H = 0.89 Å respectively for CH_2_, CH_3_, NH_3_ cation groups and O—H = 0.82 Å for the diphosphoric anion with *U*_iso_(H) = 1.5U_eq_(C, O or N). The water H atoms were refined using restraints [O—H = 0.85 (1) Å, H···H = 1.44 (2) Å and *U*_iso_(H) = 1.5U_eq_(O)].

### Crystal data

**Table:**

(C_8_H_12_NO)_2_[Mn(H_2_P_2_O_7_)_2_(H_2_O)_2_]·2H_2_O	*F*(000) = 782
*M**_r_* = 755.29	*D*_x_ = 1.615 Mg m^−^^3^
Monoclinic, *P*2_1_/*c*	Ag *K*α radiation, λ = 0.56087 Å
Hall symbol: -P 2ybc	Cell parameters from 25 reflections
*a* = 13.971 (2) Å	θ = 9–11°
*b* = 12.150 (3) Å	µ = 0.37 mm^−^^1^
*c* = 9.169 (2) Å	*T* = 293 K
β = 93.80 (4)°	Prism, colorless
*V* = 1553.0 (6) Å^3^	0.3 × 0.2 × 0.1 mm
*Z* = 2	

### Data collection

**Table:**

Enraf–Nonius CAD-4 diffractometer	4417 reflections with *I* > 2σ(*I*)
Radiation source: fine-focus sealed tube	*R*_int_ = 0.028
Graphite monochromator	θ_max_ = 28.0°, θ_min_ = 2.2°
non–profiled ω scans	*h* = −2→23
Absorption correction: multi-scan (Blessing, 1995)	*k* = −20→2
*T*_min_ = 0.920, *T*_max_ = 0.933	*l* = −15→15
9935 measured reflections	2 standard reflections every 120 min
7513 independent reflections	intensity decay: −1%

### Refinement

**Table:**

Refinement on *F*^2^	Primary atom site location: structure-invariant direct methods
Least-squares matrix: full	Secondary atom site location: difference Fourier map
*R*[*F*^2^ > 2σ(*F*^2^)] = 0.047	Hydrogen site location: inferred from neighbouring sites
*wR*(*F*^2^) = 0.112	H atoms treated by a mixture of independent and constrained refinement
*S* = 0.98	*w* = 1/[σ^2^(*F*_o_^2^) + (0.051*P*)^2^] where *P* = (*F*_o_^2^ + 2*F*_c_^2^)/3
7513 reflections	(Δ/σ)_max_ = 0.001
212 parameters	Δρ_max_ = 0.98 e Å^−^^3^
6 restraints	Δρ_min_ = −0.45 e Å^−^^3^

### Special details

**Table:**

Geometry. All e.s.d.'s (except the e.s.d. in the dihedral angle between two l.s. planes) are estimated using the full covariance matrix. The cell e.s.d.'s are taken into account individually in the estimation of e.s.d.'s in distances, angles and torsion angles; correlations between e.s.d.'s in cell parameters are only used when they are defined by crystal symmetry. An approximate (isotropic) treatment of cell e.s.d.'s is used for estimating e.s.d.'s involving l.s. planes.
Refinement. Refinement of *F*^2^ against ALL reflections. The weighted *R*-factor *wR* and goodness of fit *S* are based on *F*^2^, conventional *R*-factors *R* are based on *F*, with *F* set to zero for negative *F*^2^. The threshold expression of *F*^2^ > σ(*F*^2^) is used only for calculating *R*-factors(gt) *etc*. and is not relevant to the choice of reflections for refinement. *R*-factors based on *F*^2^ are statistically about twice as large as those based on *F*, and *R*- factors based on ALL data will be even larger.

### Fractional atomic coordinates and isotropic or equivalent isotropic displacement parameters (Å^2^)

**Table:**

	*x*	*y*	*z*	*U*_iso_*/*U*_eq_	
Mn1	0.5000	0.5000	0.5000	0.01975 (7)	
P1	0.40929 (3)	0.24580 (3)	0.52770 (4)	0.01970 (8)	
P2	0.61630 (3)	0.25518 (3)	0.48651 (4)	0.02071 (8)	
O1	0.36950 (13)	0.19825 (11)	0.38021 (12)	0.0416 (4)	
H1O1	0.3705	0.2459	0.3170	0.062*	
O2	0.40268 (9)	0.36785 (9)	0.53325 (13)	0.0254 (2)	
O3	0.36529 (9)	0.18355 (9)	0.64756 (11)	0.0265 (2)	
O4	0.51947 (10)	0.20876 (11)	0.54427 (18)	0.0434 (4)	
O5	0.68873 (10)	0.23523 (12)	0.61998 (13)	0.0372 (3)	
H5O5	0.6689	0.2648	0.6925	0.056*	
O6	0.60365 (9)	0.37401 (9)	0.45084 (14)	0.0281 (2)	
O7	0.64655 (11)	0.18277 (10)	0.36615 (12)	0.0328 (3)	
O8	0.15813 (12)	0.37510 (16)	0.4909 (2)	0.0591 (5)	
O1W	0.53579 (13)	0.49630 (11)	0.73711 (14)	0.0397 (3)	
H1W1	0.5620 (17)	0.5541 (11)	0.775 (3)	0.060*	
H2W1	0.5603 (18)	0.4381 (11)	0.776 (3)	0.060*	
O2W	0.24876 (11)	0.49273 (11)	1.02182 (16)	0.0372 (3)	
H1W2	0.2768 (17)	0.5511 (12)	1.057 (3)	0.056*	
H2W2	0.2729 (17)	0.4349 (11)	1.062 (2)	0.056*	
N1	0.27883 (11)	0.49595 (12)	0.72005 (17)	0.0308 (3)	
H1N1	0.2782	0.4801	0.8148	0.046*	
H2N1	0.3274	0.5412	0.7059	0.046*	
H3N1	0.2859	0.4342	0.6696	0.046*	
C1	0.18760 (15)	0.54946 (17)	0.6701 (3)	0.0406 (5)	
H1A	0.1913	0.5724	0.5692	0.049*	
H1B	0.1793	0.6150	0.7282	0.049*	
C2	0.10154 (15)	0.47695 (17)	0.6805 (2)	0.0378 (4)	
C3	0.0334 (2)	0.4970 (2)	0.7795 (3)	0.0602 (7)	
H3	0.0429	0.5533	0.8477	0.072*	
C4	−0.0495 (2)	0.4331 (4)	0.7777 (4)	0.0810 (11)	
H4	−0.0955	0.4468	0.8443	0.097*	
C5	−0.0629 (2)	0.3510 (3)	0.6785 (4)	0.0788 (10)	
H5	−0.1188	0.3094	0.6769	0.095*	
C6	0.00385 (19)	0.3278 (2)	0.5809 (4)	0.0623 (7)	
H6	−0.0061	0.2705	0.5144	0.075*	
C7	0.08660 (15)	0.39064 (18)	0.5820 (2)	0.0423 (5)	
C8	0.1448 (3)	0.2977 (3)	0.3750 (4)	0.0961 (13)	
H8A	0.1356	0.2257	0.4147	0.144*	
H8B	0.2004	0.2974	0.3188	0.144*	
H8C	0.0894	0.3178	0.3132	0.144*	

### Atomic displacement parameters (Å^2^)

**Table:**

	*U*^11^	*U*^22^	*U*^33^	*U*^12^	*U*^13^	*U*^23^
Mn1	0.02626 (17)	0.01297 (12)	0.02024 (13)	−0.00102 (13)	0.00333 (11)	−0.00008 (12)
P1	0.02775 (18)	0.01485 (17)	0.01671 (15)	−0.00373 (16)	0.00300 (13)	0.00009 (13)
P2	0.02652 (19)	0.01738 (17)	0.01837 (16)	0.00336 (16)	0.00264 (13)	0.00126 (14)
O1	0.0818 (11)	0.0257 (6)	0.0167 (5)	−0.0164 (7)	−0.0012 (6)	−0.0003 (5)
O2	0.0296 (6)	0.0157 (5)	0.0311 (6)	−0.0023 (4)	0.0044 (5)	−0.0019 (4)
O3	0.0400 (7)	0.0221 (5)	0.0178 (5)	−0.0083 (5)	0.0045 (5)	0.0020 (4)
O4	0.0319 (7)	0.0262 (6)	0.0733 (10)	0.0032 (5)	0.0135 (7)	0.0195 (7)
O5	0.0430 (7)	0.0460 (8)	0.0218 (5)	0.0175 (6)	−0.0033 (5)	−0.0051 (5)
O6	0.0313 (6)	0.0170 (5)	0.0370 (6)	0.0015 (4)	0.0111 (5)	0.0024 (4)
O7	0.0538 (8)	0.0244 (6)	0.0205 (5)	0.0076 (6)	0.0038 (5)	−0.0016 (4)
O8	0.0425 (9)	0.0722 (12)	0.0618 (10)	0.0020 (9)	−0.0021 (8)	−0.0262 (9)
O1W	0.0682 (10)	0.0235 (6)	0.0254 (6)	−0.0043 (7)	−0.0122 (6)	0.0004 (5)
O2W	0.0397 (7)	0.0319 (7)	0.0395 (7)	0.0032 (6)	−0.0020 (6)	0.0002 (6)
N1	0.0305 (7)	0.0275 (7)	0.0346 (7)	−0.0001 (6)	0.0043 (6)	0.0013 (6)
C1	0.0389 (11)	0.0283 (9)	0.0535 (12)	0.0026 (8)	−0.0065 (9)	0.0057 (8)
C2	0.0314 (9)	0.0397 (11)	0.0417 (10)	0.0038 (8)	−0.0019 (8)	0.0058 (8)
C3	0.0462 (13)	0.0774 (19)	0.0575 (14)	0.0148 (14)	0.0077 (11)	0.0006 (13)
C4	0.0421 (15)	0.125 (3)	0.078 (2)	0.0154 (18)	0.0190 (14)	0.037 (2)
C5	0.0373 (14)	0.093 (2)	0.105 (3)	−0.0157 (15)	−0.0083 (15)	0.049 (2)
C6	0.0424 (13)	0.0519 (15)	0.089 (2)	−0.0130 (11)	−0.0224 (13)	0.0182 (14)
C7	0.0328 (10)	0.0407 (11)	0.0515 (12)	−0.0007 (9)	−0.0107 (9)	0.0063 (9)
C8	0.079 (2)	0.114 (3)	0.092 (2)	0.026 (2)	−0.0193 (19)	−0.062 (2)

### Geometric parameters (Å, º)

**Table:**

Mn1—O2^i^	2.1389 (12)	O2W—H2W2	0.854 (9)
Mn1—O2	2.1389 (12)	N1—C1	1.476 (3)
Mn1—O6^i^	2.1749 (12)	N1—H1N1	0.8900
Mn1—O6	2.1749 (12)	N1—H2N1	0.8900
Mn1—O1W^i^	2.1987 (14)	N1—H3N1	0.8900
Mn1—O1W	2.1987 (14)	C1—C2	1.499 (3)
P1—O2	1.4868 (12)	C1—H1A	0.9700
P1—O3	1.4991 (12)	C1—H1B	0.9700
P1—O1	1.5400 (13)	C2—C3	1.380 (4)
P1—O4	1.6013 (15)	C2—C7	1.390 (3)
P2—O6	1.4883 (13)	C3—C4	1.393 (5)
P2—O7	1.4941 (13)	C3—H3	0.9300
P2—O5	1.5545 (14)	C4—C5	1.355 (5)
P2—O4	1.5886 (15)	C4—H4	0.9300
O1—H1O1	0.8200	C5—C6	1.363 (5)
O5—H5O5	0.8200	C5—H5	0.9300
O8—C7	1.357 (3)	C6—C7	1.385 (3)
O8—C8	1.422 (3)	C6—H6	0.9300
O1W—H1W1	0.855 (9)	C8—H8A	0.9600
O1W—H2W1	0.852 (9)	C8—H8B	0.9600
O2W—H1W2	0.861 (9)	C8—H8C	0.9600
			
O2^i^—Mn1—O2	180.0	C1—N1—H1N1	109.5
O2^i^—Mn1—O6^i^	86.53 (5)	C1—N1—H2N1	109.5
O2—Mn1—O6^i^	93.47 (5)	H1N1—N1—H2N1	109.5
O2^i^—Mn1—O6	93.47 (5)	C1—N1—H3N1	109.5
O2—Mn1—O6	86.53 (5)	H1N1—N1—H3N1	109.5
O6^i^—Mn1—O6	180.00 (7)	H2N1—N1—H3N1	109.5
O2^i^—Mn1—O1W^i^	87.07 (6)	N1—C1—C2	113.65 (16)
O2—Mn1—O1W^i^	92.93 (6)	N1—C1—H1A	108.8
O6^i^—Mn1—O1W^i^	94.56 (6)	C2—C1—H1A	108.8
O6—Mn1—O1W^i^	85.44 (6)	N1—C1—H1B	108.8
O2^i^—Mn1—O1W	92.93 (6)	C2—C1—H1B	108.8
O2—Mn1—O1W	87.07 (6)	H1A—C1—H1B	107.7
O6^i^—Mn1—O1W	85.44 (6)	C3—C2—C7	118.7 (2)
O6—Mn1—O1W	94.56 (6)	C3—C2—C1	122.0 (2)
O1W^i^—Mn1—O1W	180.0	C7—C2—C1	119.3 (2)
O2—P1—O3	116.66 (7)	C2—C3—C4	120.2 (3)
O2—P1—O1	112.62 (7)	C2—C3—H3	119.9
O3—P1—O1	108.22 (7)	C4—C3—H3	119.9
O2—P1—O4	109.79 (7)	C5—C4—C3	119.7 (3)
O3—P1—O4	103.12 (8)	C5—C4—H4	120.1
O1—P1—O4	105.47 (10)	C3—C4—H4	120.1
O6—P2—O7	116.38 (7)	C4—C5—C6	121.5 (3)
O6—P2—O5	112.70 (8)	C4—C5—H5	119.2
O7—P2—O5	106.76 (7)	C6—C5—H5	119.2
O6—P2—O4	109.04 (7)	C5—C6—C7	119.2 (3)
O7—P2—O4	109.02 (9)	C5—C6—H6	120.4
O5—P2—O4	101.92 (9)	C7—C6—H6	120.4
P1—O1—H1O1	109.5	O8—C7—C6	124.5 (2)
P1—O2—Mn1	134.61 (8)	O8—C7—C2	114.79 (19)
P2—O4—P1	134.68 (9)	C6—C7—C2	120.7 (3)
P2—O5—H5O5	109.5	O8—C8—H8A	109.5
P2—O6—Mn1	135.10 (8)	O8—C8—H8B	109.5
C7—O8—C8	119.1 (2)	H8A—C8—H8B	109.5
Mn1—O1W—H1W1	116.6 (16)	O8—C8—H8C	109.5
Mn1—O1W—H2W1	119.0 (16)	H8A—C8—H8C	109.5
H1W1—O1W—H2W1	111.4 (19)	H8B—C8—H8C	109.5
H1W2—O2W—H2W2	111 (2)		
			
O3—P1—O2—Mn1	136.77 (10)	O1W^i^—Mn1—O6—P2	−116.08 (12)
O1—P1—O2—Mn1	−97.20 (12)	O1W—Mn1—O6—P2	63.92 (12)
O4—P1—O2—Mn1	19.99 (13)	N1—C1—C2—C3	111.1 (2)
O6^i^—Mn1—O2—P1	−178.37 (10)	N1—C1—C2—C7	−72.7 (2)
O6—Mn1—O2—P1	1.63 (10)	C7—C2—C3—C4	−1.5 (4)
O1W^i^—Mn1—O2—P1	86.87 (11)	C1—C2—C3—C4	174.7 (2)
O1W—Mn1—O2—P1	−93.13 (11)	C2—C3—C4—C5	0.2 (4)
O6—P2—O4—P1	22.20 (18)	C3—C4—C5—C6	1.0 (5)
O7—P2—O4—P1	−105.83 (15)	C4—C5—C6—C7	−0.8 (4)
O5—P2—O4—P1	141.55 (15)	C8—O8—C7—C6	7.3 (4)
O2—P1—O4—P2	−38.38 (18)	C8—O8—C7—C2	−172.6 (2)
O3—P1—O4—P2	−163.37 (14)	C5—C6—C7—O8	179.7 (2)
O1—P1—O4—P2	83.20 (16)	C5—C6—C7—C2	−0.5 (4)
O7—P2—O6—Mn1	139.06 (11)	C3—C2—C7—O8	−178.5 (2)
O5—P2—O6—Mn1	−97.14 (12)	C1—C2—C7—O8	5.2 (3)
O4—P2—O6—Mn1	15.27 (14)	C3—C2—C7—C6	1.6 (3)
O2^i^—Mn1—O6—P2	157.14 (11)	C1—C2—C7—C6	−174.7 (2)
O2—Mn1—O6—P2	−22.86 (11)		

Symmetry code: (i) −*x*+1, −*y*+1, −*z*+1.

### Hydrogen-bond geometry (Å, º)

**Table:**

*D*—H···*A*	*D*—H	H···*A*	*D*···*A*	*D*—H···*A*
O1—H1*O*1···O3^ii^	0.82	1.77	2.5689 (17)	164
O5—H5*O*5···O7^iii^	0.82	1.76	2.5711 (18)	169
O1*W*—H1*W*1···O3^iv^	0.86 (1)	1.98 (1)	2.8304 (19)	174 (3)
O1*W*—H2*W*1···O7^iii^	0.85 (1)	2.04 (1)	2.879 (2)	167 (2)
O2*W*—H1*W*2···O7^iv^	0.86 (1)	2.03 (1)	2.886 (2)	175 (3)
O2*W*—H2*W*2···O3^iii^	0.85 (1)	2.05 (1)	2.8842 (19)	164 (2)
N1—H1*N*1···O2*W*	0.89	1.97	2.826 (2)	160
N1—H2*N*1···O6^i^	0.89	2.06	2.826 (2)	144
N1—H3*N*1···O8	0.89	2.45	2.991 (2)	120
N1—H3*N*1···O2	0.89	2.27	2.957 (2)	134

Symmetry codes: (i) −*x*+1, −*y*+1, −*z*+1; (ii) *x*, −*y*+1/2, *z*−1/2; (iii) *x*, −*y*+1/2, *z*+1/2; (iv) −*x*+1, *y*+1/2, −*z*+3/2.
